# Supplementary material for: Aedes aegypti CCEae3A carboxylase expression confers carbamate, organophosphate and limited pyrethroid resistance in a model transgenic mosquito
Source: PLoS Negl Trop Dis. 2024 Feb 20;18(2):e0011595. doi: 10.1371/journal.pntd.0011595 (PMC10906864; doi:10.1371/journal.pntd.0011595)
Supplement: S1 Data — (DOCX) [file pntd.0011595.s003.docx]

| Mosquito line | Mean CCE activity +/-SE  (μmoles/min/mg) | Welch test  P value |
| --- | --- | --- |
| Ubi-GAL4/Ubi-GAL4 | 0.018 +/-0.005 | <0.0001 |
| 3A+/3A+ | 0.47 +/-0.07 |  |

S1 Data

**Carboxylesterase activity against para-nitrophenyl acetate (PNPA) in control and 3A+/3A+ mosquitoes.**

Method:

Eight randomly picked 3-5 day old female mosquitoes from each line from 2 separate batches were assayed in duplicate readings for CCE activity using the standard WHO method for kinetic measurement of p-Nitrophenol production (1). Briefly, each mosquito was homogenised in 200μl of 50mM Sodium Phosphate buffer pH7.4, centrifuged at 15K for 30 seconds, and then 10μl added to 200ul of 1mM PNPA (diluted in 50mM Sodium Phosphate buffer pH7.4 from 100mM stock in acetonitrile). Absorbance at 405nm was then measured every 10secs for 2 mins in a Molecular Devices Sprectramax iD3 spectrophotometer. Protein concentration was measured by standard Bradford assay with Bio-Rad Quickstart reagent. CCE activity was converted from absorbance readings using an extinction co-efficient of 18000M^-1^cm^-1^ and a path length of 0.6 cm. Welch’s t-test was used to assess statistical significance.

References

1) Techniques to detect insecticide resistance mechanisms (Field and laboratory manual)

1998 Eds M. Zaim / WHOPES, WHO/CDS/CPC/MAL/98.6
